# Kinetic modeling with total body PET—current status and future applications

**DOI:** 10.1093/bjr/tqag139

**Published:** 2026-06-04

**Authors:** Beverley F Holman, Neil Davis, Adnan Chowdhury, Jannat Khan, Hemangini Natarajan, Richard Meades, Tamar Willson, Viraj Yoosoof, Daniel McCool

**Affiliations:** Department of Nuclear Medicine, Royal Free Hospital, London NW3 2QG, United Kingdom; Division of Medicine, UCL, London WC1E 6JF, United Kingdom; Department of Nuclear Medicine, Royal Free Hospital, London NW3 2QG, United Kingdom; Department of Radiation Physics and Protection, Oxford University Hospitals NHS Foundation Trust, Churchill Hospital, Headington, Oxford OX3 7LE, United Kingdom; Department of Nuclear Medicine, Royal Free Hospital, London NW3 2QG, United Kingdom; Department of Nuclear Medicine, Royal Free Hospital, London NW3 2QG, United Kingdom; Department of Nuclear Medicine, Royal Free Hospital, London NW3 2QG, United Kingdom; Department of Nuclear Medicine, Royal Free Hospital, London NW3 2QG, United Kingdom; Department of Nuclear Medicine, Royal Free Hospital, London NW3 2QG, United Kingdom; Department of Nuclear Medicine, Royal Free Hospital, London NW3 2QG, United Kingdom; Department of Nuclear Medicine, Royal Free Hospital, London NW3 2QG, United Kingdom

**Keywords:** kinetics, dynamic imaging, parametric imaging, PET/CT, LAFOV, total-body PET

## Abstract

Total-body and long axial field-of-view (LAFOV) PET/CT systems have transformed kinetic modeling from a regional research tool into a whole-body quantitative imaging approach. Unlike static SUV-based imaging, kinetic analysis provides physiologically meaningful parameters describing tracer transport, metabolism, and receptor binding. Historically limited by short axial coverage and low sensitivity, dynamic PET studies were confined to single organs and required invasive arterial sampling. LAFOV scanners, offering axial coverage of up to 2 m and >10-fold sensitivity gains, enable simultaneous multi-organ dynamic acquisitions in a single bed position. This facilitates image-derived input functions, shortened protocols, and ultra-low-dose imaging while maintaining quantitative accuracy. Applications span oncology, neurology, cardiology, infection, and inflammation, with emerging roles in healthy volunteer studies and systemic physiology research. AI-driven methods further enhance feasibility by reducing scan duration, improving parametric image generation, and automating motion correction. Despite challenges in workflow standardization and model selection, advances in acquisition protocols and computational tools position LAFOV PET kinetic modeling as a clinically viable technique. Future developments promise integration into routine practice, enabling precision diagnostics, longitudinal monitoring, and novel tracer evaluation. LAFOV PET thus represents a paradigm shift toward comprehensive, whole-body quantitative imaging in nuclear medicine.

## Introduction

PET kinetic analysis offers a powerful way to quantify biological processes in vivo, providing insights into blood flow, metabolism, receptor binding, and drug pharmacokinetics.^1^^,^^2^ Unlike static imaging based on standardized uptake values (SUVs), kinetic modeling yields parameters that relate directly to underlying physiology,^3^ providing significantly richer information about tracer behavior and tissue function. These techniques have advanced understanding of disease and treatment response across oncology, cardiology, neurology, and infection imaging.^4^^,^^5^

To date, kinetic PET has been largely restricted to research settings. Wider clinical adoption has been limited by practical challenges, including relatively low sensitivity and restricted axial coverage of 15-30 cm associated with conventional short axial field-of-view (SAFOV) systems.^1^^,^^4^ As a result, most kinetic studies focus on single organs or regions rather than the whole body.

Recent advances in detector design have led to long axial field-of-view (LAFOV) PET systems such as the Biograph Vision Quadra (Siemens)^6^ and the uEXPLORER (United Imaging)^7^ with axial lengths ranging from around 1 m to more than 2 m.^1^^,^^2^^,^^8^ These scanners combine greatly extended axial coverage with markedly higher sensitivity, achieving greater than 10- to 40-fold gains over older and more modern digital PET/CT scanners respectively.^2^^,^^4^^,^^9^ In addition they have excellent time-of-flight (TOF) performance and sub-5 mm spatial resolution.^6^^,^^7^

The combination of ultra-high sensitivity and extended AFOV provides transformative advantages for kinetic modeling.^1^^,^^2^ Entire-body dynamic acquisitions can be performed in a single bed position, enabling simultaneous multi-organ time-activity curves, image-derived input functions from large vessels, and multi-parametric modeling across the body.^10^^,^^11^ Improved signal-to-noise ratio (SNR) supports shorter frame durations, shortened total scan times, or reduced injected activities without sacrificing quantitative accuracy.^3^^,^^8^ Moreover, LAFOV coverage facilitates advanced physiological investigations such as dual-blood or multi-tracer paradigms, which are impractical on short-AFOV scanners.^12–14^

With these improvements and the development of new kinetic techniques, there is a growing opportunity to move kinetic imaging into the clinic, providing clinicians with far more detailed information than before.^3^ Consequently, LAFOV PET/CT has transitioned kinetic modeling from a regional to a whole-organism framework, enabling simultaneous quantitative assessment of tracer kinetics across multiple organs and systems.^1^^,^^2^^,^^14^ In parallel, recent work has explored abbreviated acquisition protocols and simplified input function approaches to facilitate clinical implementation.

This review outlines PET kinetic principles, before summarizing current applications in oncology, neurology, cardiology, infection and inflammation and healthy cohorts, introducing emerging applications such as artificial intelligence (AI), addressing key technical challenges and finally discussing future opportunities.

## Fundamentals of kinetic analysis in PET

Quantitative analysis of dynamic PET data provides a window into physiological and biochemical processes in vivo by modeling the time course of radiotracer concentration within tissues. Fundamentally, tissue time-activity curves (T-TACs) are described as the convolution of the arterial input function (AIF) with a tissue impulse response, allowing estimation of kinetic parameters that reflect transport and metabolism.^1^ Rigorous mathematical analysis is presented excellently elsewhere.^1–3^^,^^15^

One practical means of describing tissue kinetics is through compartmental modeling, which provides the mathematical foundation for kinetic analysis in PET.^1^^,^^2^ The simplest compartment model, the 1-tissue model (1 T), describes reversible exchange of tracer between plasma and tissue, governed by influx ($K_{1}$) and efflux ($k_{2}$) rate constants. The 2-tissue compartment model (2 T) introduces an additional compartment representing a bound or metabolized tracer fraction, described by rate constants $k_{3}$ and $k_{4}$. The irreversible 2 T model, where $k_{4}=0$, underpins glucose metabolism studies with ^18^F-FDG, yielding the net uptake rate constant $K_{i}=(K_{1}k_{3})/(k_{2}+k_{3}).$^1^^,^^3^^,^^14^^,^^15^ For reversible tracers, such as receptor or inflammation ligands, the distribution volume $V_{T}=(K_{1}/k_{2})(1+k_{3}/k_{4})$ reflects the ratio of tissue to plasma concentration at equilibrium.^1^

Model selection depends on both tracer biochemistry and tissue physiology. For instance, while the irreversible 2 T model appropriately describes ^18^F-FDG kinetics over the time course of the PET scan, reversible tracer behavior often characterizes hepatic^16^ or inflammatory uptake.^17^ In LAFOV PET studies, simultaneous multi-organ imaging introduces region-dependent kinetics that may require adaptive or hybrid model frameworks to ensure consistent parameter estimation.^1^^,^^2^^,^^12–15^

While compartmental models provide a detailed mechanistic description of tracer kinetics, they are often limited by model assumptions, noise and temporal sampling.^1–3^ Graphical analysis using Patlak^18^ and Logan^19^ offer computationally simpler alternatives for estimating macro-parameters in a more robust manner. The Patlak approach linearizes irreversible uptake to yield a slope corresponding to $K_{i}$, whereas the Logan method applies to reversible tracers to estimate $V_{T}$. These approaches reduce noise sensitivity, mitigate the impact of model mis-specification, and facilitate voxel-wise or organ-wise parametric imaging across the large datasets produced by LAFOV PET.^1–3^^,^^15^^,^^20^

For compartmental or graphical methods to yield accurate kinetic quantification, reliable knowledge of the plasma IF is essential. Arterial blood sampling remains the reference standard but is invasive and logistically complex.^11^ Advances with LAFOV PET now permit robust image-derived input functions (IDIFs) from large vessels (ie, ascending aorta or left ventricle).^1^^,^^2^^,^^10^ To further simplify protocols, population-based input functions (PBIFs) scaled by late-time image data can approximate individual arterial curves, allowing substantially shortened acquisitions without compromising $K_{i}$ accuracy.^8^^,^^10^^,^^21^

In whole-body dynamic imaging, organ-dependent delay and dispersion effects must be carefully accounted for to ensure accurate kinetic estimation. Gu et al^3^ demonstrated that uncorrected temporal offsets between blood and tissue time-activity curves can bias rate constants, particularly in high-temporal-resolution imaging of the lungs. To account for vascular heterogeneity, especially in pulmonary and tumor applications, where tissue activity reflects contributions from both arterial and venous supplies, dual-blood input modeling has been proposed in which separate input functions are used to represent these distinct vascular sources.^12^ With LAFOV PET, spatially varying bolus arrival times between IDIFs and tissue curves become increasingly relevant, potentially introducing parameter bias if uncorrected. Li et al^15^ demonstrated that delay correction, whether through joint parameter estimation or efficient pulse-timing methods, is essential for accurate voxel-wise kinetic mapping in LAFOV ^18^F-FDG PET. These correction strategies are now being integrated into quantitative pipelines to improve precision in regional and parametric imaging analyses.^1–3^^,^^14^^,^^15^^,^^20^

Gu and Wu (2023)^2^ noted that achieving accurate quantification also requires meticulous management of temporal sampling, motion, and delay effects, each of which can introduce systematic bias if uncorrected. Motion correction and gating remain essential, particularly for thoracic and abdominal studies, and ongoing methodological work continues to refine correction strategies for robust, motion-compensated whole-body kinetic modeling.^3^^,^^14^^,^^15^

## Current and future applications of kinetic analysis with total-body PET/CT

### Oncology

Kinetic analysis enables quantitative characterization of tumor biology, providing parameters that inform on processes such as metabolism, receptor binding, perfusion and proliferation.^2^ By modeling these dynamics over time, kinetic analysis yields metrics that extend beyond SUV-based imaging, offering greater insight into patient physiology and disease mechanisms. These measures are increasingly valuable, both for basic oncological research and for clinical applications such as early diagnosis, treatment selection, and therapy monitoring.

Cancer causes systemic metabolic disruption, including cognitive impairment, cachexia and insulin resistance. Tao et al^22^ performed dynamic total-body ^18^F-FDG PET in untreated lung cancer patients and healthy controls to explore inter-organ metabolic connectivity, finding markedly reduced network coupling in cancer, suggesting system-wide metabolic dysregulation. The high sensitivity and extended coverage of LAFOV PET/CT make such analyses feasible by improving signal-to-noise and enabling concurrent multi-organ kinetic assessment.

Kinetic PET has a well-established role in evaluating new radiopharmaceuticals, and the introduction of LAFOV systems has accelerated this. Omidvari et al^23^ quantified ^18^F-F-AraG uptake, a tracer for activated T-cells, to assess immune cell infiltration within the tumor microenvironment and predict immunotherapy response. Dynamic LAFOV studies with ^18^F-PSMA-1007^24^ or with ^68^Ga-PSMA-11^25^ have enabled assessment of both organ and tumor pharmacokinetics, applying compartmental models to derive quantitative parameters. In the latter, parametric imaging revealed tumor heterogeneity not visible on SUV imaging, underscoring the added diagnostic value of voxel-wise kinetic data.

Several studies have demonstrated improved lesion contrast and characterization through kinetic parameters. Meng et al^26^ showed that combining blood-volume fraction (*V*_b_), net influx rate ($K_{i}$) and SUV provided better discrimination of pulmonary lesions than SUV alone. Similarly, Wu et al^27^ found that ^18^F-FDG metabolic rate ($K_{i}$) images in lung cancer exhibited higher contrast-to-noise ratio (CNR) than SUV images; in one case, a suspected lesion was excluded on $K_{i}\;$imaging and later confirmed as benign. Wu et al^28^ further showed that whole-body parametric images of ^18^F-FDG kinetics could be generated with scan times as short as 10 minutes while retaining quantitative accuracy, albeit with slightly increased noise and with an additional radiation dose. The group uses 2 protocols, one which requires either an additional low-dose CT for registration purposes or an additional administration of ^18^F-FDG. Finally, parametric $V_{T}$ images of ^68^Ga-FAPI-04 improved lesion contrast in pancreatic and gastric cancer,^29^ while ^18^F-FDG and ^68^Ga-PSMA-11 kinetics analysis of $K_{i}$ and $k_{3}$ yielded superior tumor to background ratios and higher reader confidence.^25^^,^^30^ Patlak parametric imaging has also shown advantages for subtle lesion detection. Pan et al^30^ found that despite overall lesion detectability between SUV and Patlak imaging remaining comparable, in 1 case Patlak imaging was able to reveal small liver metastases that were obscured in conventional SUV imaging. In Chen et al,^25^ the study reported that through the use of $K_{i}$ images, they were able to identify additional lesions both visible in SUV images, including lymph node and bone metastases, leading to changes in therapeutic management highlighting potential added value in certain clinical scenarios. The study also demonstrated the ability of kinetic images, specifically receptor binding/internalisation rate ($k_{3}$) in distinguishing between pathological prostate cancer lesions and normal physiological uptake outperforming SUV, $k_{1}$, $k_{2}$ and $K_{i3}$ parameters.

An important consideration when adopting parametric imaging is its clinical application. The studies above have generally shown that the primary benefit from parametric imaging is from improved lesion contrast and detectability rather than parameter accuracy. Introducing protocols with either shortened acquisition times or simplified input functions are acceptable. However, there may be applications that require more precise parameter estimation such as therapy response monitoring or longitudinal research to ensure measured changes reflect biological changes rather than due to technical variability.

While ^18^F-FDG remains the most extensively studied tracer for kinetics, recent work demonstrates the broad potential of LAFOV dynamic imaging across multiple radiopharmaceuticals. Pedersen et al^31^ showed that dynamic whole-body ^18^F-FES PET in metastatic breast cancer improved lesion visibility and correlation with full kinetic analysis, aiding estrogen receptor classification. Similarly, dynamic ^68^Ga-FAPI and PSMA imaging has provided improved differentiation between malignant and benign uptake patterns and facilitated pharmacokinetic profiling for therapy planning.^25^^,^^29^ Early-dynamic ^18^F-FDG imaging has further been extended to estimate blood flow using distributed kinetic models validated against ^11^C-butanol,^32^ suggesting that LAFOV PET can enable simultaneous flow-metabolism assessment with a single tracer.

These developments illustrate how kinetic imaging with LAFOV PET/CT can characterize both tumor biology and systemic physiology, potentially bridging oncological, cardiovascular and metabolic domains.

### Neurology

Neurological PET imaging remains a fundamental component in many disease pathways including Alzheimer’s and Parkinson’s.^33^ Kinetic modeling can be used to extract clinically useful parameters such as the non-displaceable binding potential (BP_ND_) which is considered more robust to changes in cerebral blood flow. However, its use has so far been limited by the need for arterial sampling and lengthy scan durations which can be difficult for the aging population.

The carotid artery is typically used to derive the IDIF, however it is susceptible to partial volume effects (PVE) due to their small diameter of ∼5mm. An increased FOV can address the PVE by enabling evaluation of IDIF accuracy by acquiring data from larger blood pools.^11^ Holy et al^34^ evaluated the use of descending aorta-based IDIFs to extract BP_ND_ values with and without arterial blood sampling in 15 patients imaged with ^18^F-Florbetapen. As ^18^F-Florbetapen is a reversible tracer, pseudo-equilibrium cannot be assumed, making measurements such as the BP_ND_ more useful than the SUV, which would otherwise overestimate the amount of ^18^F-Florbetapen present. They identified BP_ND_ values could be used to reliably distinguish between amyloid positive and negative groups which may support earlier intervention. A similar study of 15 patients performed by Bhattarai et al^35^ using ^18^F-PI-2620, found that kinetic parameters could be accurately established in the absence of arterial blood sampling. However other studies suggest the role of arterial sampling may still be required. A study performed by Salvi de Souza et al^36^ investigated IDIFs from the aortic arch and the carotid artery for ^18^F-MC225, a tracer used to image P-glycoprotein (P-gp) function at the blood-brain barrier, in 6 patients. They found the calibrated carotid artery IDIF provided consistent results with minimal bias and low variability, however this still required the technique to be calibrated using arterial sampling. Alternatively, other methods could be used to address the limitations of using a carotid artery IDIF. Zhu et al^37^ utilized an LAFOV scanner to develop a kernel simultaneous estimation framework (SIME) which estimated the blood input function and kinetic parameters for ^18^F-FDG brain imaging. They demonstrated better estimates of the blood input function in comparison to using the carotid artery IDIF.

The superior properties of a LAFOV scanner also enable noninvasive brain perfusion estimations to be made which can provide further insight into tracer behavior. This has been demonstrated by Larsson et al^38^ who used a Tikhonov generalized deconvolution to investigate whether brain perfusion could be estimated using the descending aorta for the IF for 5 different PET tracers. They found this method could provide reliable estimations of perfusion regardless of how the tracer moves across the blood-brain barrier.

Collectively, these developments highlight how LAFOV PET enables more robust and less invasive quantification of neuroreceptor binding and cerebral perfusion, addressing key methodological barriers that have historically limited the clinical application of kinetic modeling in neurological imaging. However, most studies remain limited to small cohorts, and further validation is required to establish clinical impact and routine applicability.

### Cardiology

A key advantage of LAFOV PET for cardiovascular kinetic modeling is that it extends quantitative assessment beyond the conventional cardiac field-of-view, allowing myocardial blood flow (MBF), vascular activity, and extracardiac organ physiology to be assessed in the same acquisition.^4^^,^^39^ Although MBF quantification is already well established with conventional cardiac PET, the main added value of LAFOV is simultaneous evaluation of myocardial perfusion alongside systemic vascular and multi-organ kinetics. This is particularly relevant to coronary microvascular dysfunction and systemic inflammatory or infiltrative disease, where cardiac abnormalities may coexist with renal, neurological, vascular, or metabolic involvement.^40–42^ However, the clinical impact of this approach remains under investigation, with most current evidence derived from feasibility and small-cohort studies.

^15^O-water remains the reference tracer for perfusion quantification because it is freely diffusible and has a near-linear relationship with blood flow. Rainio et al^43^ evaluated 6 1TCM variants in dynamic total-body ^15^O-water PET across 20 anatomical regions in 58 subjects using an aortic IDIF. Their model performance was organ-dependent: a single aortic 1TCM was suitable for several regions, including the myocardium, brain, spleen, and kidneys, but performed poorly in the liver and lung lobes, leading the authors to recommend alternative approaches for hepatic and pulmonary blood-flow quantification. This is consistent with the greater modeling complexity of these organs including dual hepatic blood supply, lung air-volume, and pulmonary vessel inclusion within lung VOIs.^44^^,^^45^ More recently, total body ^15^O-water PET during adenosine stress showed organ-specific perfusion responses, strengthening the rationale for systemic rather than isolated cardiac assessment.^46^ Despite these advantages, the approximately 2-minute half-life of ^15^O and requirement for on-site cyclotron production limit routine implementation in centers without suitable radiochemistry infrastructure.

Clinically practical LAFOV perfusion approaches may therefore rely on generator-produced or longer-lived tracers. A proof-of-concept rest-stress dynamic whole-body ^82^Rb LAFOV study showed that myocardial and renal flow could be derived from the same acquisition, illustrating how established PET perfusion tracers may be adapted for systemic cardiovascular assessment.^47^ 18F-Flurpiridaz is another promising option, with high first-pass myocardial extraction, favorable image quality, and phase 3 evidence supporting CAD assessment; however, UK clinical translation will depend on licensing, availability, commissioning and cost-effectiveness pathways.^48^^,^^49^ 18F-FDG has also been explored for total-body blood-flow imaging using high temporal resolution early dynamic LAFOV PET; Chung et al^50^ showed strong overall correlation with ^11^C-butanol flow estimates, but patient-specific differences suggest that further validation is required.

Beyond perfusion, LAFOV PET may strengthen vascular and infiltrative cardiac imaging. Dynamic ^18^F-FDG can generate Patlak-derived arterial-wall metabolic-rate images with improved lesion detectability compared with SUV,^51^ while total-body ^18^F-FDG PET enables high-contrast vessel-wall imaging at delayed timepoints as blood-pool activity falls.^52^ This supports late static imaging as a practical complement to kinetic modeling, particularly for low-signal vascular inflammation.

Overall, future applications are likely to focus on simultaneous multi-organ assessment, including amyloid burden, FAPI-based imaging of myocardial and extracardiac fibrosis, and human validation of neurocardiac and cardiorenal inflammatory interactions.^53–56^ While these developments highlight the potential of LAFOV PET for integrated cardiovascular and systemic assessment, further standardization and validation are required before routine clinical adoption.

### Infection and inflammation

LAFOV kinetic studies have helped understand the nature of long COVID. Wang et al^57^ conducted dynamic ^18^F-FDG scans of 13 healthy controls and 12 patients recovering from COVID. They found that while SUV and CT number were not significantly different between groups, there were differences in kinetic parameters that revealed effects of long COVID. In lung tissue, $K_{i}$ was significantly higher in the COVID convalescent group, showing the superior ability of kinetics at detecting differences in glucose metabolism. While $k_{1}$ was similar in both groups, the lower $k_{3}$ in the recovering cohort indicates that these variations are due to differences in phosphorylation. Conversely, within the bone marrow, $k_{1}$ was significantly higher in the COVID group, indicating a difference in glucose delivery. Within the COVID convalescent group, the $K_{i}$ was lower in the lungs and higher in the spleen in the vaccinated than the unvaccinated group. Kinetic studies therefore give more valuable insights into the mechanism of long COVID and the protective value of vaccination when compared to static PET.

Other tracers have been used to investigate immune system mechanisms more directly. Omidvari et al^23^ used ^89^Zr labeled CD8-targetted minibodies to investigate the biodistribution of CD8+ T cells in 5 patients post-COVID infection and three healthy volunteers. They concluded that tissue-to-blood ratios and $K_{i}$ were greater in the COVID convalescent group than in the control group. Kinetic analysis provided less invasive and a higher precision method for estimating this than flow cytometry,^58^ making quantitation in tissues other than blood more viable. The ability to track immune cells over time demonstrates the value of kinetic analysis in studying immune response and memory.

Whole-body ^18^F-FDG kinetics has been explored as a noninvasive tool for assessing the extent of liver inflammation in patients with nonalcoholic fatty liver disease (NAFLD). In a study of 14 healthy subjects and 10 patients with NAFLD,^59^ they found that both $k_{1}$ and the interstitial distribution volume were significantly lower in patients that had a higher inflammation score than those with a lower score and healthy volunteers.

Dynamic imaging can help determine the presence of infection as distinct from inflammation alone in ^18^F-FDG studies.^60^ In a study of 31 patients with fractures, van Rijsewijk et al^60^ observed that in the 14 patients with clinically confirmed infection the SUV rose consistently during the 65 minutes of the scan, whereas in the patients without infection the SUV plateaued. Further analysis and kinetic modeling may help in future patient management.

### Healthy volunteer studies

Quantitative PET studies in healthy volunteers are invaluable for defining normal physiological ranges, validating kinetic models, and establishing baseline tracer pharmacokinetics. Yet despite this importance, such data remain extremely limited, largely due to radiation exposure associated with conventional PET imaging. Traditional PET systems require relatively high administered activities to achieve adequate count statistics for kinetic studies, resulting in radiation doses that regularly preclude ethical recruitment of healthy participants. Consequently, true whole-body kinetic datasets in healthy volunteers are rare. The advent of LAFOV PET/CT scanners fundamentally changes this paradigm.

LAFOV PET/CT enables substantial dose reduction through its high sensitivity, allowing reliable kinetic analysis at lower administered activities. Liu et al^61^ conducted ultra-low-dose dynamic LAFOV PET/CT imaging in 12 healthy volunteers, injecting activities of 0.37 MBq/kg with a median effective dose of 0.42 mSv. This was a 10-fold reduction in activity from the full-dose cohort scanned, while maintaining comparable kinetic constant rates and image contrast. Yin et al^62^ extended this approach to receptor imaging, performing dynamic LAFOV scans with ^68^Ga-DOTATATE, injecting activities of only 1 MBq/kg resulting in a median effective dose of 1.78 mSv, half their standard clinical dose, while successfully quantifying kinetic parameters across multiple normal organs.

In contrast to dose reduction, which impacts radiation exposure, LAFOV PET also enables abbreviated acquisition protocols that improve workflow and patient tolerability without compromising kinetic accuracy. Feng et al^63^ first demonstrated the feasibility of total-body parametric imaging using only the initial 90 s of post-injection ^18^F-FDG data in healthy volunteers, providing similar estimates of $k_{1}$ and blood fraction parameters when compared to the full 60-minute acquisition. Liu et al^64^ demonstrated this potential in 11 healthy volunteers using LAFOV ^18^F-FDG PET with injected activities of approximately 108 MBq, showing that reliable rate constants and Patlak slopes could be derived even with shortened (45 minutes) acquisitions. Most recently, Chung et al^32^ conducted early-dynamic ^18^F-FDG imaging in 34 healthy participants, acquiring 1- to 2-second temporal frames to model blood flow and vascular transit times noninvasively.

Collectively, these studies illustrate that LAFOV PET has transformed healthy-volunteer kinetic research from aspiration to reality. By enabling low-dose, whole-body, multi-organ, shorter duration dynamic imaging, LAFOV PET provides a foundation for normative kinetic atlases, improved tracer validation, and cross-organ physiological modeling.

### AI and computation methods

Recent advances in AI are increasingly being investigated as tools to enhance the capabilities of LAFOV PET/CT imaging.^65^ Zhang et al^66^ highlight how AI, with its ability to process large volumes of high-dimensional data, can help manage the growing data demands and complexity associated with LAFOV PET/CT, with dynamic parametric imaging identified as a key area of application. Similarly, Wang et al^67^ discuss how developments in machine learning (ML) and deep learning (DL) offer promising strategies to address specific challenges associated with LAFOV kinetic modeling.

For example, DL methods combined with kinetic models have been used to generate 1 hour equivalent dynamic images using only 30 minutes of dynamic data.^68^ Similar approaches have shown that dynamic acquisitions can be generated from dual-time-point protocols. These demonstrate the potential for parametric image estimation with reduced scan times and improved patient comfort.^69^

Another area of progress is parametric image generation from static SUV images, limited dynamic frames or combinations of both. Building on earlier discussions of abbreviated acquisition protocols and simplified input function approaches across different clinical applications, DL and ML models have demonstrated the ability to estimate parametric maps from limited data and without explicit IF’s, offering significant reductions in acquisition time and avoiding blood sampling while preserving quantification accuracy,^3^^,^^70–73^ thereby extending these clinically motivated strategies for reducing acquisition burden. DL-based denoising has been shown to improve image quality and reduce variability in parameter estimates, supporting the feasibility of lower-dose protocols.^74^

Motion correction and segmentation are additional challenges where DL approaches have shown promise. DL methods for inter-frame motion correction, including unsupervised^75^ and model-informed approaches,^76^ achieve faster and more accurate alignment than conventional non-rigid registration, improving reliability of parametric fitting. ML clustering-based segmentation approaches are being explored for dynamic LAFOV datasets, though further refinement is needed to achieve clinically robust performance.^77^

For all these potential methods, generalizability of AI tools remains a key limitation to adoption, with performance degrading across scanners, institutions and patient populations particularly where training data are homogeneous, camera and protocol specific. Any AI methods developed for LAFOV kinetic modeling will require rigorous validation against established approaches, ideally using external multicenter datasets. Furthermore, clinical implementation must align with evolving regulatory frameworks for software and AI as a medical device, requiring clear clinical utility, reproducibility, transparency, and ongoing performance monitoring.

## Technical challenges and standardization

Translating kinetic analysis into routine clinical practice faces challenges of accessibility, workflow, and standardization.^25^^,^^26^^,^^78^^,^^79^ Motion and delay corrections significantly affect kinetic accuracy, particularly in multi-input organs such as the liver.^79^ Organ segmentation and time-activity curve extraction remain variable across centers and software platforms. Once obtained, model selection introduces further heterogeneity; even for the same tracer, optimal compartmental models can differ between tumor types and individuals.^25^^,^^26^^,^^78^^,^^79^ Voxel-wise compartmental modeling has been proposed to address this issue. Wang et al^80^ demonstrated that incorporating voxel-specific delay correction and model selection improved quantitative accuracy in LAFOV ^18^F-FDG imaging, reducing vascular artifacts and enhancing lesion quantification. Such approaches exemplify how model adaptability may reconcile biological heterogeneity with methodological consistency.^25^

Efforts to streamline kinetic imaging using LAFOV PET have focused on reducing scan duration and computational burden. Viswanath et al^81^ showed that abbreviated 10-15 minutes early dynamic acquisitions followed by a short delayed scan accurately reproduced full 60-minute ^18^F-FDG kinetics. Wu et al^28^ and Liu et al^82^ confirmed that shortened or dual-injection protocols could yield $K_{i}$ maps comparable to full-length studies, suggesting feasible workflow integration. Perfusion-related constants such as $k_{1}$ can even be estimated from the first 90 seconds of data using LAFOV systems.^32^^,^^63^ These optimized protocols address key clinical constraints while maintaining quantitative integrity.

Dose reduction is another important avenue. Alberts et al^9^ and Dimitrakopoulou-Strauss et al^83^ reported that LAFOV scanners maintain diagnostic image quality at activities < 1 MBq/kg and enable ultra-low-dose or rapid scanning expanding applicability for longitudinal monitoring and pediatric imaging.

For any new imaging approach to enter routine clinical use, it must demonstrate tangible clinical utility that justifies its cost. In PET, this value is defined by improved lesion detection, characterization or therapy response assessment that changes management. Although long-axial-field-of-view (LAFOV) PET/CT systems entail greater initial and operational costs, their substantially higher sensitivity and temporal resolution can shorten acquisitions, enable lower doses and deliver richer quantitative data.^9^^,^^84^ Advances in dynamic acquisition protocols and reconstruction now permit clinically feasible kinetic imaging within minutes, often improving reader confidence.^25^^,^^78^^,^^81^ With ongoing optimization and AI-assisted standardization,^30^ the enhanced quantitative insight offered by kinetic modeling is increasingly likely to justify its adoption, positioning LAFOV PET/CT as a transformative rather than costly addition to precision oncology.

## Future applications

Despite rapid technical advances, the current evidence base for LAFOV PET kinetic modeling remains limited. Most studies to date are small, single-center feasibility investigations, with relatively little quantitative evidence demonstrating impact on diagnostic performance, clinical decision-making, or patient outcomes. While improvements in lesion detectability, contrast, and physiological insight have been consistently reported, the generalizability of these findings remains uncertain, and robust multicenter validation is still required. As such, the clinical role of kinetic modeling in total-body PET should currently be considered investigational, with significant potential but limited outcome-based evidence to support widespread routine adoption.

The future of kinetic analysis with LAFOV PET lies in translating whole-body quantitative imaging into practical, clinically meaningful tools. Fully dynamic acquisitions may soon be incorporated into standard patient slots, enabling routine generation of parametric maps that augment or surpass SUV-based assessment. Simplified kinetic models, robust IDIFs, and AI-driven parameter estimation will reduce reliance on long acquisitions and arterial sampling, making quantification feasible in clinical workflows.

Ultra-low-dose and short-duration protocols will expand access for pediatric and frail patients and facilitate longitudinal monitoring with minimal dose. Parametric imaging of metabolism, perfusion, and receptor availability may allow earlier detection of disease in oncology, neurology, cardiology, and inflammatory disorders, leading to earlier treatment.

In research, whole-body simultaneous kinetic imaging will enable new insights into cross-organ physiology, systemic disease networks, and drug distribution. Tracers previously impractical due to short half-lives, low uptake, or complex kinetics may become viable again.

Overall, LAFOV PET kinetic analysis has the potential to transform quantitative imaging from a specialized research tool into a core component of precision medicine.

## Conclusion

LAFOV PET/CT has redefined the landscape of kinetic imaging, transforming it from a regional to a whole-organism science. Its unparalleled sensitivity and coverage enable low-dose, high-temporal-resolution dynamic studies. Healthy-volunteer and multi-organ physiological research can now become a reality. These advances unlock opportunities for standardized biomarkers, improved tracer validation, and precision diagnostics.

Future progress depends on integrating AI, automated analysis, and harmonized protocols to fully realize the potential of LAFOV kinetic modeling.

## References

[1] Rahmim A , Lodge MA, Karakatsanis NA, et al Dynamic whole-body PET imaging: principles, potentials and applications. Eur J Nucl Med Mol Imaging. 2019;46:501-518. 10.1007/s00259-018-4153-6 30269154

[2] Gu F , Wu Q. Quantitation of dynamic total-body PET imaging: recent developments and future perspectives. Eur J Nucl Med Mol Imaging. 2023;50:3538-3557. 10.1007/s00259-023-06299-w 37460750 PMC10547641

[3] Gu W , Zhu Z, Liu Z, et al An improved Patlak-based K_i_ parametric imaging approach for clinical^18^ F-FDG total-body PET. Phys Med Biol. 2024;70:015017. 10.1088/1361-6560/ad9ce4 39657322

[4] Cherry SR , Diekmann J, Bengel FM. Total-body positron emission tomography. JACC Cardiovasc Imaging. 2023;16:1335-1347. 10.1016/j.jcmg.2023.06.022 37676207

[5] Karakatsanis NA. Long axial field-of-view (LAFOV) PET in the era of multi-parametric imaging and theranostics. Br J Radiol. 2026;13:tqag060. 10.1093/bjr/tqag060 41830147

[6] Prenosil GA , Sari H, Fürstner M, et al Performance characteristics of the biograph vision quadra PET/CT system with a long axial field of view using the NEMA NU 2-2018 standard. J Nucl Med. 2022;63:476-484. 10.2967/jnumed.121.261972 34301780

[7] Spencer BA , Berg E, Schmall JP, et al Performance evaluation of the uEXPLORER total-body PET/CT scanner based on NEMA NU 2-2018 with additional tests to characterize PET scanners with a long axial field of view. J Nucl Med. 2021;62:861-870. 10.2967/jnumed.120.250597 33008932 PMC8729871

[8] Sari H , Eriksson L, Mingels C, et al Feasibility of using abbreviated scan protocols with population-based input functions for accurate kinetic modeling of [18F]-FDG datasets from a long axial FOV PET scanner. Eur J Nucl Med Mol Imaging. 2023;50:257-265. 10.1007/s00259-022-05983-7 36192468 PMC9816288

[9] Alberts I , Sari H, Mingels C, et al Long-axial field-of-view PET/CT: perspectives and review of a revolutionary development in nuclear medicine based on clinical experience in over 7000 patients. Cancer Imaging. 2023;23:28. 10.1186/s40644-023-00540-3 36934273 PMC10024603

[10] Palard-Novello X , Visser D, Tolboom N, et al Validation of image-derived input function using a long axial field of view PET/CT scanner for two different tracers. EJNMMI Phys. 2024;11:25. 10.1186/s40658-024-00628-0 38472680 PMC10933214

[11] Volpi T , Maccioni L, Colpo M, et al An update on the use of image-derived input functions for human PET studies: new hopes or old illusions? EJNMMI Res. 2023;13:97. 10.1186/s13550-023-01050-w 37947880 PMC10638226

[12] Wang Y , Abdelhafez YG, Spencer BA, et al High-temporal-resolution kinetic modeling of lung tumors with dual-blood input function using total-body dynamic PET. J Nucl Med. 2024;65:714-721. 10.2967/jnumed.123.267036 38548347 PMC11064825

[13] Li L , Wan L, Zhao H, Wang C, Wei W, Liu J. Biodistribution and radiation dosimetry of multiple tracers on total-body positron emission tomography/computed tomography. Quant Imaging Med Surg. 2023;13:5182-5194. 10.21037/qims-22-1418 37581077 PMC10423372

[14] Wang Y , Spencer BA, Schmall J, et al High-temporal-resolution lung kinetic modeling using total-body dynamic PET with time-delay and dispersion corrections. J Nucl Med. 2023;64:1154-1161. 10.2967/jnumed.122.264810 37116916 PMC10315691

[15] Li EJ , Spencer BA, Schmall JP, et al Efficient delay correction for total-body PET kinetic modeling using pulse timing methods. J Nucl Med. 2022;63:1266-1273. 10.2967/jnumed.121.262968 34933888 PMC9364346

[16] Wang J , Shao Y, Liu B, et al Dynamic 18F-FDG PET imaging of liver lesions: evaluation of a two-tissue compartment model with dual blood input function. BMC Med Imaging. 2021;21:90. 10.1186/s12880-021-00623-2 34034664 PMC8152049

[17] Zoghi S , Mingels C, Badawi RD, et al Role of total body PET/CT in inflammatory disorders. Semin Nucl Med. 2025;55:41-51. 10.1053/j.semnuclmed.2024.11.001 39578110 PMC11645246

[18] Patlak CS , Blasberg RG. Graphical evaluation of blood-to-brain transfer constants from multiple-time uptake data. Generalizations. J Cereb Blood Flow Metab. 1985;5:584-590. 10.1038/jcbfm.1985.87 4055928

[19] Logan J , Fowler JS, Volkow ND, et al Graphical analysis of reversible radioligand binding from time—activity measurements applied to [*N*-^11^ C-methyl]-(−)-cocaine PET studies in human subjects. J Cereb Blood Flow Metab. 1990;10:740-747. 10.1038/jcbfm.1990.127 2384545

[20] Yang Q , Li W, Huang Z, et al Bidirectional dynamic frame prediction network for total-body [68Ga]Ga-PSMA-11 and [68Ga]Ga-FAPI-04 PET images. EJNMMI Phys. 2024;11:92. 10.1186/s40658-024-00698-0 39489859 PMC11532329

[21] Sari H , Mingels C, Alberts I, et al First results on kinetic modelling and parametric imaging of dynamic 18F-FDG datasets from a long axial FOV PET scanner in oncological patients. Eur J Nucl Med Mol Imaging. 2022;49:1997-2009. 10.1007/s00259-021-05623-6 34981164

[22] Tao X , Wang H, Qu J, et al Exploring the metabolic landscape of lung adenocarcinoma and squamous cell carcinoma: a total-body [18F]FDG PET/CT approach. Eur J Nucl Med Mol Imaging. 2025;53:697-701. 10.1007/s00259-025-07371-3 40658198

[23] Omidvari N , Jones T, Price PM, et al First-in-human immunoPET imaging of COVID-19 convalescent patients using dynamic total-body PET and a CD8-targeted minibody. Sci Adv. 2023;9:eadh7968. 10.1126/sciadv.adh7968 37824612 PMC10569706

[24] Sachpekidis C , Pan L, Groezinger M, Strauss DS, Dimitrakopoulou-Strauss A. Combined whole-body dynamic and static PET/CT with low-dose [18F]PSMA-1007 in prostate cancer patients. Eur J Nucl Med Mol Imaging. 2024;51:2137-2150. 10.1007/s00259-024-06620-1 38286936 PMC11139746

[25] Chen R , Ng YL, Yang X, et al Comparison of parametric imaging and SUV imaging with [68 Ga]Ga-PSMA-11 using dynamic total-body PET/CT in prostate cancer. Eur J Nucl Med Mol Imaging. 2024;51:568-580. 10.1007/s00259-023-06456-1 37792025

[26] Meng N , Zhang M, Ren J, et al Quantitative parameters of static imaging and fast kinetics imaging in 18F-FDG total-body PET/CT for the assessment of histological feature of pulmonary lesions. Quant Imaging Med Surg. 2023;13:5579-5592. 10.21037/qims-23-186 37711783 PMC10498229

[27] Wu Y , Fu F, Meng N, et al The role of dynamic, static, and delayed total-body PET imaging in the detection and differential diagnosis of oncological lesions. Cancer Imaging. 2024;24:2. 10.1186/s40644-023-00649-5 38167538 PMC10759379

[28] Wu Y , Feng T, Zhao Y, et al Whole-Body parametric imaging of^18^ F-FDG PET using uEXPLORER with reduced scanning time. J Nucl Med. 2022;63:622-628. 10.2967/jnumed.120.261651 34385335 PMC8973287

[29] Chen R , Yang X, Ng YL, et al First total-body kinetic modeling and parametric imaging of dynamic^68^ Ga-FAPI-04 PET in pancreatic and gastric cancer. J Nucl Med. 2023;64:960-967. 10.2967/jnumed.122.264988 36604180

[30] Pan L , Sachpekidis C, Hassel J, Christopoulos P, Dimitrakopoulou-Strauss A. Impact of different parametric Patlak imaging approaches and comparison with a 2-tissue compartment pharmacokinetic model with a long axial field-of-view (LAFOV) PET/CT in oncological patients. Eur J Nucl Med Mol Imaging. 2025;52:623-637. 10.1007/s00259-024-06879-4 39256215 PMC11732916

[31] Pedersen MA , Munk OL, Dias AH, et al Dynamic whole-body [18F]FES PET/CT increases lesion visibility in patients with metastatic breast cancer. EJNMMI Res. 2024;14:24. 10.1186/s13550-024-01080-y 38436824 PMC10912074

[32] Chung KJ , Chaudhari AJ, Nardo L, et al Quantitative total-body imaging of blood flow with high-temporal-resolution early dynamic 18F-FDG PET kinetic modeling. J Nucl Med. 2025;66:973-980. 10.2967/jnumed.124.268706 40306973 PMC12175980

[33] Villemagne VL , Barkhof F, Garibotto V, Landau SM, Nordberg A, Van Berckel BNM. Molecular imaging approaches in dementia. Radiology. 2021;298:517-530. 10.1148/radiol.2020200028 33464184 PMC7924525

[34] Holy EN , Li E, Alfaro ER, et al Non‐invasive kinetic modeling of [^18^ F]‐florbetaben and [^18^ F]‐PI‐2620 with total‐body dynamic EXPLORER PET. Alzheimers Dement. 2023;19:e075087. 10.1002/alz.075087

[35] Bhattarai A , Holy EN, Wang Y, et al Kinetic modeling of 18F-PI-2620 binding in the brain using an image-derived input function with total-body PET. EJNMMI Res. 2025;15:62. 10.1186/s13550-025-01260-4 40445477 PMC12125444

[36] Salvi De Souza G , Mossel P, Somsen JF, et al Evaluating image-derived input functions for cerebral [18F]MC225 PET studies. Front Nucl Med. 2025;5:1597902. 10.3389/fnume.2025.1597902 40538986 PMC12176838

[37] Zhu Y , Tran Q, Wang Y, et al Optimization-derived blood input function using a kernel method and its evaluation with total-body PET for brain parametric imaging. Neuroimage. 2024;293:120611. 10.1016/j.neuroimage.2024.120611 38643890 PMC11251003

[38] Larsson HBW , Law I, Andersen TL, et al Brain perfusion estimation by Tikhonov model-free deconvolution in a long axial field of view PET/CT scanner exploring five different PET tracers. Eur J Nucl Med Mol Imaging. 2024;51:707-720. 10.1007/s00259-023-06469-w 37843600 PMC10796558

[39] Slart RHJA , Bengel FM, Akincioglu C, et al Total-Body PET/CT applications in cardiovascular diseases: a perspective document of the SNMMI cardiovascular council. J Nucl Med. 2024;65:607-616. 10.2967/jnumed.123.266858 38388512

[40] Konst RE , Guzik TJ, Kaski JC, Maas AHEM, Elias-Smale SE. The pathogenic role of coronary microvascular dysfunction in the setting of other cardiac or systemic conditions. Cardiovasc Res. 2020;116:817-828. 10.1093/cvr/cvaa009 31977015 PMC7526753

[41] Ishiyama M , Soine LA, Vesselle HJ. Semi-quantitative metabolic values on FDG PET/CT including extracardiac sites of disease as a predictor of treatment course in patients with cardiac sarcoidosis. EJNMMI Res. 2017;7:67. 10.1186/s13550-017-0315-y 28822108 PMC5561746

[42] Hong Z , Xue S, Yu J, et al Total-body 11C-PIB PET/CT imaging of systemic amyloidosis: inter-organ connectivity in cardiac amyloidosis for prognostic insights. Eur J Nucl Med Mol Imaging. 2025;52:4985-4999. 10.1007/s00259-025-07308-w 40323422 PMC12589323

[43] Rainio O , Knuuti J, Klén R. Investigation of total-body applicable compartment models for kinetic modeling in total body ^15^O-water perfusion PET imaging. Netw Model Anal Health Inform Bioinforma. 2025;14:22. 10.1007/s13721-025-00515-3

[44] Rainio O , Knuuti J, Klén R. New compartment model for hepatic blood flow quantification in humans from 15O-water PET images. Eur J Nucl Med Mol Imaging. 2025;52:3500-3509. 10.1007/s00259-025-07210-5 40116877 PMC12222372

[45] Rainio O , Kärpijoki H, Knuuti J, Klén R. Pulmonary blood flow quantification in humans from 15O-water PET. Ann Nucl Med. 2025;39:600-607. 10.1007/s12149-025-02035-6 40087214 PMC12095458

[46] Kärpijoki H , Tuisku J, Palonen S, et al Organ-specific perfusion response to adenosine as measured using total-body PET. J Nucl Med. 2026;26:jnumed.125.271613. 10.2967/jnumed.125.271613 PMC13321948 41748294

[47] Caobelli F , Seibel S, Krieger K, et al First-time rest-stress dynamic whole-body 82Rb-PET imaging using a long axial field-of-view PET/CT scanner. Eur J Nucl Med Mol Imaging. 2023;50:2219-2221. 10.1007/s00259-023-06242-z 37103564 PMC10199881

[48] Maddahi J , Agostini D, Bateman TM, et al Flurpiridaz F-18 PET myocardial perfusion imaging in patients with suspected coronary artery disease. J Am Coll Cardiol. 2023;82:1598-1610. 10.1016/j.jacc.2023.08.016 37821170

[49] Keam SJ. Flurpiridaz F 18: first approval. Am J Cardiovasc Drugs. 2025;25:1-6. 10.1007/s40256-024-00718-5 39862355

[50] Chung KJ , Chaudhari AJ, Nardo L, et al Quantitative total-body imaging of blood flow with high-temporal-resolution early dynamic^18^ F-FDG PET kinetic modeling. J Nucl Med. 2025;66:973-980. 10.2967/jnumed.124.268706 40306973 PMC12175980

[51] Derlin T , Werner RA, Weiberg D, Derlin K, Bengel FM. Parametric imaging of biologic activity of atherosclerosis using dynamic whole-body positron emission tomography. JACC Cardiovasc Imaging. 2022;15:2098-2108. 10.1016/j.jcmg.2022.05.008 36481078

[52] Derlin T , Spencer BA, Mamach M, et al Exploring vessel wall biology in vivo by ultrasensitive total-body PET. J Nucl Med. 2023;64:416-422. 10.2967/jnumed.122.264550 36175139 PMC10071799

[53] Santarelli MF , Genovesi D, Scipioni M, et al Cardiac amyloidosis characterization by kinetic model fitting on [18F]florbetaben PET images. J Nucl Cardiol. 2022;29:1919-1932. 10.1007/s12350-021-02608-8 33864226

[54] Cui Y , Wang Y, Wang S, Du B, Li X, Li Y. Highlighting fibroblasts activation in fibrosis: the state-of-the-art fibroblast activation protein inhibitor PET imaging in cardiovascular diseases. J Clin Med. 2023;12:6033. 10.3390/jcm12186033 37762974 PMC10531835

[55] Thackeray JT , Hupe HC, Wang Y, et al Myocardial inflammation predicts remodeling and neuroinflammation after myocardial infarction. J Am Coll Cardiol. 2018;71:263-275. 10.1016/j.jacc.2017.11.024 29348018

[56] Werner RA , Hess A, Koenig T, et al Molecular imaging of inflammation crosstalk along the cardio-renal axis following acute myocardial infarction. Theranostics. 2021;11:7984-7994. 10.7150/thno.61423 34335975 PMC8315063

[57] Wang Y , Nardo L, Spencer BA, et al Total-Body multiparametric PET quantification of^18^ F-FDG delivery and metabolism in the study of coronavirus disease 2019 recovery. J Nucl Med. 2023;64:1821-1830. 10.2967/jnumed.123.265723 37591539 PMC10626370

[58] Robinson JP , Ostafe R, Iyengar SN, Rajwa B, Fischer R. Flow cytometry: the next revolution. Cells. 2023;12:1875. 10.3390/cells12141875 37508539 PMC10378642

[59] Tran Q , Matsukuma K, Spencer B, et al High-temporal resolution kinetic modeling on total-body PET differentiates the hepatic interstitial space in nonalcoholic fatty liver disease and healthy subjects. J Nucl Med. 2022;63:2518.

[60] Van Rijsewijk ND , Wouthuyzen-Bakker M, Van Snick JH, et al Clinical evaluation of dynamic [18F]FDG PET imaging to distinguish infection from inflammation in fracture-related infections. Eur J Nucl Med Mol Imaging. 2026;53:2167-2177. 10.1007/s00259-025-07563-x 40974409 PMC12860805

[61] Liu G , Hu P, Yu H, et al Ultra-low-activity total-body dynamic PET imaging allows equal performance to full-activity PET imaging for investigating kinetic metrics of 18F-FDG in healthy volunteers. Eur J Nucl Med Mol Imaging. 2021;48:2373-2383. 10.1007/s00259-020-05173-3 33479842

[62] Yin H , Liu G, Hu Y, et al Dynamic total-body PET/CT imaging reveals kinetic distribution of 68Ga-DOTATATE in normal organs. Contrast Media Mol Imaging. 2023;2023:4722499. 10.1155/2023/4722499 36713636 PMC9876673

[63] Feng T , Zhao Y, Shi H, et al Total-body quantitative parametric imaging of early kinetics of^18^ F-FDG. J Nucl Med. 2021;62:738-744. 10.2967/jnumed.119.238113 32948679 PMC8844261

[64] Liu G , Yu H, Shi D, et al Short-time total-body dynamic PET imaging performance in quantifying the kinetic metrics of 18F-FDG in healthy volunteers. Eur J Nucl Med Mol Imaging. 2022;49:2493-2503. 10.1007/s00259-021-05500-2 34417855

[65] Shiyam Sundar LK , Gutschmayer S, Maenle M, Beyer T. Extracting value from total-body PET/CT image data - the emerging role of artificial intelligence. Cancer Imaging. 2024;24:51. 10.1186/s40644-024-00684-w 38605408 PMC11010281

[66] Zhang Q , Huang Z, Jin Y, et al Total-Body PET/CT: a role of artificial intelligence? Semin Nucl Med. 2025;55:124-136. 10.1053/j.semnuclmed.2024.09.002 39368911

[67] Wang Y , Li E, Cherry SR, Wang G. Total-body PET kinetic modeling and potential opportunities using deep learning. PET Clin. 2021;16:613-625. 10.1016/j.cpet.2021.06.009 34353745 PMC8453049

[68] Liang G , Zhou J, Chen Z, et al Combining deep learning with a kinetic model to predict dynamic PET images and generate parametric images. EJNMMI Phys. 2023;10:67. 10.1186/s40658-023-00579-y 37874426 PMC10597982

[69] Ding W , Wang H, Qiao X, Li B, Huang Q. A deep learning method for total-body dynamic PET imaging with dual-time-window protocols. Eur J Nucl Med Mol Imaging. 2025;52:1448-1459. 10.1007/s00259-024-07012-1 39688700

[70] Miao T , Zhou B, Liu J, et al Generation of whole-body FDG parametric *K*_i_ images from static PET images using deep learning. IEEE Trans Radiat Plasma Med Sci. 2023;7:465-472. 10.1109/TRPMS.2023.3243576 37997577 PMC10665031

[71] Huang Z , Wu Y, Fu F, et al Parametric image generation with the uEXPLORER total-body PET/CT system through deep learning. Eur J Nucl Med Mol Imaging. 2022;49:2482-2492. 10.1007/s00259-022-05731-x 35312030

[72] Zaker N , Haddad K, Faghihi R, Arabi H, Zaidi H. Direct inference of Patlak parametric images in whole-body PET/CT imaging using convolutional neural networks. Eur J Nucl Med Mol Imaging. 2022;49:4048-4063. 10.1007/s00259-022-05867-w 35716176 PMC9525418

[73] Li Y , Hu J, Sari H, et al A deep neural network for parametric image reconstruction on a large axial field-of-view PET. Eur J Nucl Med Mol Imaging. 2023;50:701-714. 10.1007/s00259-022-06003-4 36326869

[74] Muller FM , Li EJ, Daube-Witherspoon ME, et al Impact of deep learning denoising on kinetic modelling for low-dose dynamic PET: application to single- and dual-tracer imaging protocols. Eur J Nucl Med Mol Imaging. 2025;52:3465-3483. 10.1007/s00259-025-07182-6 40069458 PMC12222255

[75] Guo X , Zhou B, Pigg D, et al Unsupervised inter-frame motion correction for whole-body dynamic PET using convolutional long short-term memory in a convolutional neural network. Med Image Anal. 2022;80:102524. 10.1016/j.media.2022.102524 35797734 PMC10923189

[76] Guo X , Zhou B, Chen X, Chen MK, Liu C, Dvornek NC. MCP-Net: introducing Patlak loss optimization to whole-body dynamic PET inter-frame motion correction. IEEE Trans Med Imaging. 2023;27:3512-3523. 10.1109/TMI.2023.3290003 PMC10751388 37368811

[77] Jaakkola MK , Rantala M, Jalo A, et al Segmentation of dynamic total-body [18F]-FDG PET images using unsupervised clustering. Int J Biomed Imaging. 2023;2023:3819587. 10.1155/2023/3819587 38089593 PMC10715853

[78] Wang Z , Wu Y, Li X, et al Comparison between a dual-time-window protocol and other simplified protocols for dynamic total-body 18F-FDG PET imaging. EJNMMI Phys. 2022;9:63. 10.1186/s40658-022-00492-w 36104580 PMC9474964

[79] Moradi H , Vashistha R, O’Brien K, et al Model selection for dynamic PET compartmental modelling of 18F-FDG uptake using a long axial field-of-view PET scanner with delay and motion correction. EJNMMI Res. 2025;15:81. 10.1186/s13550-025-01277-9 40613830 PMC12227408

[80] Wang G , Nardo L, Parikh M, et al Total-body PET multiparametric imaging of cancer using a voxelwise strategy of compartmental modeling. J Nucl Med. 2022;63:1274-1281. 10.2967/jnumed.121.262668 34795014 PMC9364337

[81] Viswanath V , Sari H, Pantel AR, et al Abbreviated scan protocols to capture 18F-FDG kinetics for long axial FOV PET scanners. Eur J Nucl Med Mol Imaging. 2022;49:3215-3225. 10.1007/s00259-022-05747-3 35278108 PMC10695012

[82] Liu G , Shi Y, Hou X, et al Dynamic total-body PET/CT imaging with reduced acquisition time shows acceptable performance in quantification of [18F]FDG tumor kinetic metrics. Eur J Nucl Med Mol Imaging. 2024;51:1371-1382. 10.1007/s00259-023-06526-4 38078950

[83] Dimitrakopoulou-Strauss A , Pan L, Sachpekidis C. Parametric imaging with dynamic PET for oncological applications: protocols, interpretation, current applications and limitations for clinical use. Semin Nucl Med. 2022;52:312-329. 10.1053/j.semnuclmed.2021.10.002 34809877

[84] Dimitrakopoulou-Strauss A , Pan L, Sachpekidis C. Total body PET-CT protocols in oncology. Semin Nucl Med. 2025;55:3-10. 10.1053/j.semnuclmed.2024.05.008 38851935

